# Neuronal Roles of the Multifunctional Protein Dipeptidyl Peptidase-like 6 (DPP6)

**DOI:** 10.3390/ijms23169184

**Published:** 2022-08-16

**Authors:** Cole Malloy, Maisie Ahern, Lin Lin, Dax A. Hoffman

**Affiliations:** Molecular Neurophysiology and Biophysics Section, Eunice Kennedy Shriver National Institute of Child Health and Human Development, National Institutes of Health, Bethesda, MD 20892, USA

**Keywords:** DPP6, Kv4.2, voltage-gated potassium channel, A-type K^+^ channel, neuronal excitability, neuronal development, synaptogenesis

## Abstract

The concerted action of voltage-gated ion channels in the brain is fundamental in controlling neuronal physiology and circuit function. Ion channels often associate in multi-protein complexes together with auxiliary subunits, which can strongly influence channel expression and function and, therefore, neuronal computation. One such auxiliary subunit that displays prominent expression in multiple brain regions is the Dipeptidyl aminopeptidase-like protein 6 (DPP6). This protein associates with A-type K^+^ channels to control their cellular distribution and gating properties. Intriguingly, DPP6 has been found to be multifunctional with an additional, independent role in synapse formation and maintenance. Here, we feature the role of DPP6 in regulating neuronal function in the context of its modulation of A-type K^+^ channels as well as its independent involvement in synaptic development. The prevalence of DPP6 in these processes underscores its importance in brain function, and recent work has identified that its dysfunction is associated with host of neurological disorders. We provide a brief overview of these and discuss research directions currently underway to advance our understanding of the contribution of DPP6 to their etiology.

## 1. Introduction

Dipeptidyl aminopeptidase-like protein 6 (DPP6/DPPX/DPL1/BSPL/potassium channel accelerating factor, KAF), is a type II transmembrane protein first isolated and cloned from bovine and rat brain tissue [1,2]. DPP6 derives from a ubiquitous family of serine peptidases crucial for normal cell function in prokaryotes and eukaryotes. Included in this family are DPP4 (DPPIV/CD26), DPP8 (DP8), DPP9 (DP9), and DPP10 (DPL2/DPPY). Both DPP8 and DPP9 are involved in an inflammatory form of cell death [3,4], while DPP4 is notable for its role in diabetic and cancer therapies [5]. Unlike other members of the family, both DPP6 and DPP10 (together referred to as DPPLs) have a critical enzymatic serine to aspartate substitution, rendering their protease function inactive [6,7,8,9]. Interest in potential alternative functions of these two proteins has been driven as a result of this observed lack of peptidase activity [6].

The DPP6 protein structure is highly similar to the antigenic exopeptidase family member DPP4 (32% sequence identity and 50% sequence similarity) [10]. Its sequence similarity with the additional family member lacking enzymatic activity, DPP10, is even greater (51% amino acid sequence identity), with features alluding to shared cellular distribution and function [11]. They contain multiple glycosylation sites and significantly higher sequence similarities (92%) in transmembrane domains. Indeed, both DPP6 and DPP10 are found in the cytoplasm and at the cell membrane, with prominent expression in various brain regions, including the hippocampus, a structure of the brain important for memory formation [11]. Intriguingly, DPP6 and DPP10 display differential expression patterns within given regions of the brain, suggesting distinctive roles in neural circuit function [11,12,13].

**Figure 1:**
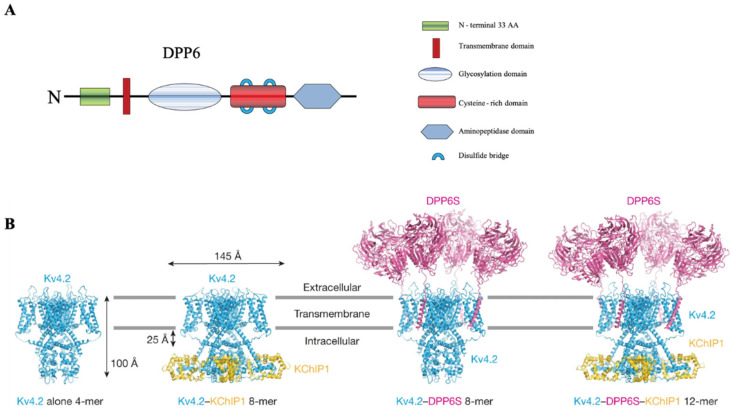
**DPP6 structure.** (**A**) Domain structures of DPP6. From Figure 1 in Lin et al., 2014 [7]. (**B**) Overall structures of the Kv4.2-alone tetramer, Kv4.2–KChIP1 octamer, Kv4.2–DPP6 octamer and Kv4.2–DPP6–KChIP1 dodecamer (left to right). Four Kv4.2 subunits are colored blue, four KChIP1 subunits are colored yellow, and four DPP6 subunits are colored magenta. Figure 1A in Kise et al., 2021 licensed under CC BY 4.0.

The gene that encodes DPP6 is located at chromosome 7q36.2 [9]. Three alternate isoforms of DPP6 in the adult rodent brain have been found: DPP6-K, DPP6-S and DPP6-L. DPP6-S expresses at highest levels, followed by DPP6-K and DPP6-L [12]. DPP6-S and DPP6-K exist predominately in hippocampal pyramidal neurons in the CA1-CA3 regions. DPP6-L, however, is mostly confined to the olfactory bulb and cerebellum [12]. They are each comprised of a short variable N-terminal and an extensive C-terminal with three distinct domains [12] (Figure 1). The first domain in the C-terminal of DPP6-S contains an eight-bladed β-propeller and an α/β hydrolase domain facilitating dimerization and houses seven n-glycosylation sites [6,14,15]. These β-propellers have been identified as important both to the structure and functioning as an oxidoreductase, which may contribute to its likely differential redox sensitivity. The second region found along the middle blades is a cysteine-rich portion, comparable to DPP4 (DPPIV/CD26). Similar to DPP4, four disulfide bonds important to protein folding, and subsequent transportation out of the ER, as well as its localization to the membrane are found in the second domain. Lastly, the third domain contains the Asp for Ser substitution, resulting in a loss of peptidase activity [6,7]. Consistent with other type II transmembrane proteins, DPP6 exists as a monomer with the ability to form heterodimers [2,12,16].

Insights into the non-proteolytic function of DPP6 were driven by an unexpected finding: co-precipitation with a prominent voltage-gated K^+^ channel [17]. Specifically, DPP6 co-precipitates with the Kv4 family of voltage-gated K+ channels, which have a crucial role in regulating neuronal excitability, firing patterns, and long-term potentiation (LTP) induction in the hippocampus. Kv4 channels conduct an A-type K^+^ current (I_A_), which is rapidly activating and inactivating. Using various DPP6 mutants, Lin et al. 2014 showed that the cysteine-rich domain of the C-terminus is not required to interact with Kv4.2; however, without it, DPP6 is not transported out of the ER. Experiments mutating the N-terminal, however, show that the intracellular portion is necessary for the interaction between DPP6 and Kv4.2 [7,18].

Here, we review the described functions of DPP6, focusing on its roles in neuronal excitability as a powerful regulating auxiliary subunit of A-type voltage-gated K^+^ channels and its independent role in regulating synaptic development and function. By augmenting the Kv4.2 channel conductance, DPP6 prevents hyperexcitability of neurons as well as backpropagation of action potentials [19,20,21]. More recently, novel functions of DPP6 have been brought to light. Apart from its role in influencing Kv4.2 currents, DPP6 has been identified to promote and maintain filopodia growth and stability. Using a variety of experimental techniques, Lin et al. found that DPP6 is required for filopodia to develop normally and properly transition into dendritic spines [22]. DPP6′s function in filopodia regulation ultimately leads to a critical role in behavior and brain development [23]. This can especially be seen in the supporting findings that DPP6 is associated with neurodegenerative diseases and cognitive disorders such as amyotrophic lateral sclerosis, dementia, intellectual disability, Tourette’s syndrome, microcephaly, and autism spectrum disorder in addition to others [24,25,26,27,28].

## 2. DPP6 Regulation of A-Type K^+^ Channels

### 2.1. The Search for A-Type K^+^ Channel Auxiliary Subunits

The majority of work on DPP6 has centered on its role as an auxiliary subunit of A-type K^+^ channels. These channels underlie the fast-transient, subthreshold K^+^ current (I_A_), which is a vital regulator of the intrinsic excitability of neurons in invertebrate and vertebrate species. Alpha subunits Kv1.1 (when in combination certain auxiliary subunits such as Kvb1), Kv1.4, Kv3.3, Kv3.4, Kv4.1, Kv4.2, and Kv4.3 form the full complement of pore forming proteins capable of mediating I_A_ in the mammalian brain [29,30]. While each subunit is expressed in various neuronal subtypes, the majority of neuronal I_A_ is carried by Kv4.2 and Kv4.3-containing channels [17,31,32]. As a result, a considerable amount of effort has gone into understanding the role of these channels in neuronal physiology and disease. Due to their high expression, somatodendritic localization, and subthreshold range of activation (allowing control of synaptic integration) and the pacing of action potential (AP) firing [30,33,34,35,36,37,38,39], the molecular mechanisms underlying their regulation have been intensely studied.

A particular incentive to identify auxiliary subunits affecting Kv4 conductance came about as early recordings of neuronal I_A_ found that its voltage-dependent and kinetic properties did not match that of currents measured in non-neuronal cells expressing Kv4 alpha subunits (Maffie and Rudy 2008). Initial insights were provided over two decades ago when An et al. (2000) [40] identified K^+^ channel interacting proteins (KChIPs), in complex with Kv4.3, using a yeast two-hybrid screen. KChIPs are small (188–285 aa) Ca^2+^-binding proteins of the neuronal Ca^2+^ sensor (NCS) gene family expressed from four genes (KCNIP1-4). This work was pivotal in providing evidence of Kv4 channels existing in macromolecular complexes to drive native I_A_ [40]. Subsequent analyses in heterologous expression systems revealed KChIPs 1–4 exert a robust influence on Kv4 channel expression and gating kinetics. While these findings uncovered a key element of Kv4 channel regulation in neurons, there was a persistent ambiguity: Kv4 channels in complex with KChIPs produced I_A_ that was still discernibly distinctive from native currents [38,40,41]. This prompted further pursuits for Kv4 modifying subunits underlying endogenous neuronal I_A_.

### 2.2. DPP6 Is Part of a Ternary Protein Complex Regulating Neuronal Kv4-Mediated I_A_

Final recapitulation of native I_A_ was accomplished by Nadal and colleagues when Kv4 complexes were purified from rat cerebellar membranes. Among a collection of proteins pulled down via immunopurification of Kv4 was DPPX (DPP6) [10]. Evidence of the association between Kv4.2 subunits and DPP6 was strengthened by immunohistochemistry experimentation in rat brain tissue. Anti-DPP6 antibody staining revealed strong expression in neuronal populations that are highly enriched in both Kv4.2 and Kv4.3 mRNA [31,42,43]. Some of these neurons included granule cells of the cerebellar cortex, the dentate gyrus, and the olfactory bulb, and in thalamic relay neurons in thalamic nuclei. Expression was found in both neurons characterized by high Kv4.2 expression, including CA1 pyramidal neurons of the hippocampus, neurons in the pontine nucleus, the olfactory tubercle, and the striatum, and in neurons enriched for Kv4.3 expression, including Purkinje cells neurons of the substantia nigra and periglomerular area in the olfactory bulb [10]. Further, DPP6 displayed a somatodendritic subcellular distribution in hippocampal CA1 pyramidal neurons and granule cells of the dentate gyrus, which mirrors that of Kv4.2 and Kv4.3, respectively, [38,42,43,44,45], suggesting DPP6 and Kv4 channel colocalization in multiple brain regions.

**Figure 2:**
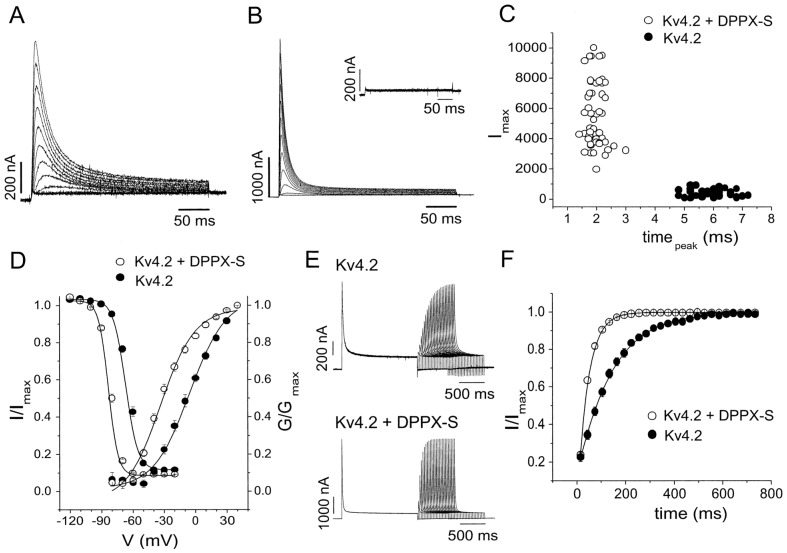
**Effects of DPP6 on Kv4.2-Mediated A-Type K^+^ Currents.** (**A**,**B**) A-type K^+^ currents recorded in *Xenopus* Oocytes with Kv4.2 expression alone (**A**) or Kv4.2 and DPP6 (**B**). (**C**) Peak current at +40 mV (I_max_) and time-to-peak (time_peak_) for the currents recorded with Kv4.2 alone or Kv4.2 plus DPP6. (**D**) Normalized conductance-voltage (G/G_max_) and steady-state inactivation (I/I_max_) curves for A-type currents with Kv4.2 alone or Kv4.2 plus DPP6. The curves were fitted with Boltzmann functions. (**E**) Recovery from inactivation of the A-type currents with Kv4.2 alone or Kv4.2 plus DPP6. Shown are the currents recorded during test pulses to 50 mV following a test pulse to the same voltage separated by increasing time intervals at −110 mV. (**F**) Time course of the recovery from inactivation of the A-type currents with Kv4.2 alone or Kv4.2 coexpressed with DPP6 at -110 mV. The curves were fitted with a single exponential. Reprinted with permission from Nadal et al., 2003 [10], Elsevier.

Importantly, the co-expression of DPP6 cRNAs with Kv4.2 and Kv4.3 in *Xenopus* oocytes and CHO cells dramatically altered Kv4-mediated I_A_. Specifically, DPP6 significantly boosted whole-cell current density, decreased the time to current peak, accelerated the rate of macro current inactivation, produced a ~28 mV negative shift in the voltage-dependence of activation, and dramatically increased the rate of recovery from inactivation relative to that observed with Kv4 expression alone [10,46] (Figure 2). Nadal et al. (2003) confirmed DPP6 is required for this alteration in current density and gating kinetics, as utilization of antisense oligonucleotides directed against DPP6 peptides abolished the DPP6-induced tuning of I_A_ and confirmed Kv4 specificity for this effect [10]. Furthermore, the expression of DPP6, Kv4.2 or 4.3, and KChIP1 together exhibited currents with properties mimicking notably fast kinetics of I_A_ native to neurons enriched in Kv4.2 or 4.3 [31]. These formative studies provided definitive evidence that DPP6 associates with Kv4 pore-forming subunits and, together with KChIPs, form a ternary macromolecular complex underlying endogenous I_A_ [17,47].

### 2.3. DPP6 Increases Macro I_A_ by Boosting Kv4 Forward Trafficking and Unitary Conductance

The observable boost in I_A_ current density in the presence of DPP6 sparked interest in the mechanisms underlying this effect. It has been established that voltage-gated channel auxiliary subunits may assist in increasing membrane expression by promoting their trafficking from the endoplasmic reticulum (ER). These subunits often mask ER retention motifs and/or assist in the proper folding of the pore-forming subunits, thereby facilitating their transfer from the ER and insertion and stabilization in the plasma membrane [48,49]. Initial studies identifying the DPP6 modulation of Kv4-mediated currents in heterologous expression systems were key in confirming that both DPP6 and DPP10 are critical in Kv4 trafficking and membrane stabilization. Nadal and colleagues (2003) examined the subcellular distribution of Kv4.2 proteins in CHO cells in the presence or absence of DPP6. They found Kv4.2 proteins concentrate within the perinuclear ER in the absence of DPP6 but redistribute to the cell surface in its presence [10]. The quantification of this effect via surface biotinylation showed 19.5 times more surface expressed Kv4.2 protein with DPP6 co-expression relative to cells transfected with Kv4.2 alone [10]. Zagha et al. [11] expanded on these findings and confirmed a similar effect on Kv4.2 channels by DPP10. Here, they created chimeric DPP6 and 10 proteins via the replacement of extracellular domains with a series of Myc tags to investigate the structural regions of DPPLs crucial in Kv4 trafficking. The chimeric proteins, which consisted of only the DPPL intracellular and transmembrane domains and lacked the entire hydrolase domain and beta-propeller, were capable of effectively trafficking Kv4.2 to the cell surface in a manner similar to full length DPPLs, suggesting that these domains are sufficient to assist in Kv4 membrane localization [11].

Lin and colleagues co-expressed Kv4.2 with multiple DPP6 constructs, consisting of a series of deletions, in HEK293 and COS7 cells. Using co-immunoprecipitation, they identified the short intracellular N-terminal domain (32 aa at the N-terminus) plus the transmembrane domain (total 54 aa) as important in facilitating DPP6-Kv4.2 interaction, while the extracellular C-terminal domain is required for trafficking out of the ER and membrane expression of Kv4.2 [7]. This confirmed previous analyses by Jerng et al. [50] as well as Ren et al. [18], who used chimeric proteins to identify a region in DPP10 from its N-terminus to the end of its transmembrane domain as responsible for its interaction with the S1 and S2 domains in the Kv4.3 voltage sensor. However, intriguingly, deletions in these domains did not significantly alter the ability of DPP6 to boost Kv4.2 surface expression above basal levels, as indicated by surface biotinylation and whole-cell voltage-clamp recordings. Rather, an increase in Kv4.2 membrane expression and current density was regulated by an extracellular, cysteine-rich domain, which Lin et al. (2014) identified as required for DPP6 trafficking out of the ER. Mutations in either of two disulfide bridges (C349/C356 and C465/C468) in this region abolished the ability of DPP6 to increase Kv4.2 surface expression and enhanced its retention in the ER. This showed that, as for DPP6 itself, the cysteine-rich domain plays a critical role in allowing forward trafficking of Kv4.2 out of the ER, even though it is not necessary for their interaction. Further, this cysteine-rich extracellular domain was integral in promoting Kv4.2 membrane stability [7]. Collectively, these findings revealed important features regarding DPPL regulation of Kv4 channels, including: (1) that the primary interaction between DPPLs and Kv4 subunits is via the DPPL N-terminal + transmembrane domains and the Kv4 S1 and S2 transmembrane voltage sensor domains and (2) there is a required role for the cysteine-rich extracellular domain in permitting Kv4 forward trafficking and membrane stability.

Recent cryo-EM studies have strengthened the notion that the DPP6 transmembrane domain facilitates modulation of Kv4 gating (Figure 1B). Kise et al. (2021) compared cryo-EM structures of the Kv4.2 tetramer alone to an octamer complex with Kv4.2 expressed along with a KChIP subunit (Kv4.2–KChIP1), an octamer complex of Kv4.2 and the short form of DPP6 (Kv4.2–DPP6S) and a Kv4.2–DPP6S–KChIP1 dodecamer complex [51]. Interestingly, DPP6S addition to Kv4.2 or Kv4.2–KChIP1 complexes resolved two different classes of structures with either two or four DPP6S molecules integrated in the complex (one or two dimers). DPP6S was found to interact with the S1 and S2 transmembrane helixes of the Kv4.2 voltage-sensing domain in the open state [51,52]. They also report that KChIP1 and DPP6S do not directly interact with each other in the Kv4.2–KChIP1–DPP6S dodecamer complex. KChIPs and DPPLs may therefore independently or additively affect Kv4 channel expression and function. Similar findings were reported soon after for Kv4.3, showing the same interactions for KChIP and DPP6 subunits [52]. One major difference appears to be that while in the Kv4.2–KChIP1–DPP6S structure, Kv4.2 complexes with either four DPP6S or two DPP6S subunits. The Kv4.3–KChIP1–DPP6 structure only complexes with two DPP6 molecules. Further study will need to determine whether these differences are due to basic differences between Kv4.2 and Kv4.3 or to some other distinctions between the studies (e.g., the use of different DPP6 isoforms). Regardless, these fundamental studies have clarified the mechanism of the crucial but distinct roles KChIPs and DPPLs play in the regulation of Kv4 channel gating.

In addition to increasing the membrane localization of Kv4 channels, DPP6 also induces larger macro I_A_ by boosting channel unitary conductance (γ). It had been noted that an additional oddity in Kv4-mediated I_A_ measured in expression systems, in the absence of auxiliary subunits, was a reduction in single-channel conductance. Reports of γ on the order 6–8.5 pS for A-type K^+^ channels in multiple neuronal subtypes had been recorded [38,53,54], which represented a 50–100% increase relative to those recorded in non-neuronal cells [55,56,57]. An elegant study by Kaulin et al. [58] addressed this ambiguity by seeking to determine if DPP6 modifies Kv4 γ. They recorded single-channel currents in tsA-201 cells expressing Kv4.2 or Kv4.2 + DPP6s and in dissociated cerebellar granule neurons (CGNs) known to express these proteins. Unitary currents from recombinant Kv4.2 channels measured between 2 and 4.1 pS, depending on holding potential, in the absence of DPP6s. With DPP6s co-expression, Kv4.2 γ increased to 5.5–7.7 pS, representing a nearly two-fold increase at peak. Importantly, the γ measured in CGNs (~7.3 pS) mirrored that of recombinant Kv4.2 + DPP6s, suggesting DPP6 is part of the native Kv4 complex in these cells. Furthermore, to confirm the requirement of DPP6 in driving intrinsic CGN currents, the group measured unitary currents in CGN cells from DPP6 knockout (DPP6-KO) mice. Channels in these neurons yielded γ of ~4.2 pS, indistinguishable from that of Kv4.2 channels, alone, in tsA-201 cells [58]. Further analysis confirmed a similar effect on Kv4.3 γ and no further changes were observed in the presence of KChIP1, suggesting this KChIP does not influence Kv4 conductance, consistent with previous studies [56,57].

In light of these findings, Kaulin et al. [58] sought to determine the mechanism underlying the favorable effect of DPP6 on Kv4 conductance. They hypothesized that negatively charged, acidic residues (D18, E20) in the juxtamembrane cytoplasmic N-terminal region of DPP6s were likely to be key. Potentially analogous negatively charged residues in the cytoplasmic alpha-subunits of large-conductance Ca^2+^-activated K^+^ channels (BK) confer their relatively robust γ, as they help to concentrate K^+^ near the inner mouth of the channel (Brelidze et al., 2003). Indeed, charge neutralization mutations affecting these two amino acids (D18N, E20Q) abolished DPP6-induced increase in Kv4.2 γ without impacting additional macroscopic properties [58]. The confirmation of this effect in multiple host cells and with Kv4.3 co-expression provided convincing evidence that these residues are integral in DPP6 modulation of Kv4 channel conductance by promoting electrostatic attraction of K^+^ to the channel pore [58]. Therefore, in addition to strongly promoting Kv4 forward trafficking and membrane stability, Kaulin and colleagues established that DPP6-induced enhancement of Kv4 γ also contributes to its boosting of macroscopic I_A._

### 2.4. DPP6 (and 10) Accelerates Kv4 Activation and Inactivation Gating

Rapid activation and inactivation are a fundamental property of Kv4 channels in neurons. Perhaps the most striking difference in early studies of KChIP regulation of Kv4 currents in expression systems was the marked extension of macro current inactivation they induced when in complex with Kv4 alpha subunits. Time constants of Kv4 current inactivation in the presence of multiple KChIPs measured 3–4× larger than those recorded in their absence [40]. Further, neuronal I_A_ decay time constants recorded with standard protocols are often described by the sum of two or three exponentials, with the fast component ranging from 40–70 ms [38,59,60,61,62,63], a ~3-fold reduction relative to KChIP-Kv4 current decay in heterologous expression systems [64]. While Kv4 channel inactivation involves many processes, including contribution by both the N-terminus and C-terminus [55,65,66], evidence indicated that fast inactivation, in the absence of auxiliary subunits, involved occlusion of the channel pore by the N-terminus [67]. The presence of KChIPs must, then, interfere with this process. The means by which this occurred were unraveled when studies identified the Kv4 cytoplasmic N-terminus (~40 residue hydrophobic segment) and a loop in the T1 tetramerization domain’s cytoplasmic face as critical in mediating Kv4-KChIP interaction [68]. The determination of the three-dimensional crystal structure of the KChIP1-Kv4.3 complex further refined our understanding. Pioletti et al. [68] determined the contact between the Kv4 T1 domain and the KChIP H2 helix to be responsible for modulation of channel gating. Additionally, a hydrophobic segment of the N-terminus of Kv4.3 is buried within a hydrophobic pocket formed by KChIP1 upon binding. This interface facilitates the functional sequestration of the Kv4 N-terminus and prevents rapid decay via N-type inactivation, a defining characteristic of other transient voltage-gated K^+^ channels [67]. Consistent with this supposition, Kv4 subunits with the N-terminus deleted (2Δ40), expressed without KChIPs, pass currents that exhibit slower inactivation kinetics, similar to complexes comprising KChIP auxiliary subunits [21,66].

Subsequently, it was shown that the association of DPP6 in the complex underlies the rapid inactivation typically observed in neurons. Formative observations by Jerng et al. [50] demonstrated that the DPPL N-terminus was critical in recouping the fast component of inactivation in Kv4 complexes. Co-expression of both DPP6 and DPP10 with Kv4.2 in *Xenopus* oocytes accelerated macro current rise time and reduced the time constant of fast macroscopic current decay by ~25% and ~60%, respectively, relative to Kv4.2 expression alone. The effect of both DPPLs on inactivation was abolished by disrupting the DPPL N-terminus. Furthermore, the creation of chimeric proteins, through the interchanging of DPP6 and 10 N-termini, demonstrated that these domains were sufficient to alter current decay. DPP6 chimeras containing DPP10 N-termini induced DPP10-like decay kinetics, with the converse effect apparent with DPP10 chimeras [50].

The mechanism by which the DPPL N-terminus modulates Kv4 inactivation was further identified in an additional study by Jerng et al. [69]. Here, the authors expressed DPP10a, DPP6a, and DPP6s isoforms together with Kv4.2 (+KChIP3) in *Xenopus* oocytes and found that DPP10a and DPP6a confer similar fast inactivation to the complex. Genomic analysis revealed a shared N-terminal motif encoded in Exon1a-MNQTA, which is conserved from fish to humans. Deletions and point mutations in this motif altered the ability of DPP10a to accelerate the fast component of macroscopic inactivation. Furthermore, the perfusion of soluble MNQTA peptide to the cytoplasmic face of Kv4.2 channels in inside-out patches reduced current amplitude by ~63% [69]. These data show that the conserved MNQTA motif in the DPP10a and DPP6a N-termini is responsible for imparting fast inactivation and may occur via a pore-blocking mechanism. Because N-type inactivation is characterized by the blockade the inner channel pore upon depolarization by an N-terminal domain, the ability of soluble MNQTA peptides to promote internal pore block suggested DPPLs may recover this ability in ternary Kv4 complexes. Jerng et al. (2009) [69] tested this directly using standard criteria indicative of classic N-type inactivation: (1) competition between tetramethylammonium (TEA), a fast open-channel blocker, and the internal domain for the occlusion site, and (2) a “knock-off” effect induced by elevated external K^+^ concentrations during recovery from inactivation [70,71]. The group used inside-out patch-clamp recordings to examine the effect of TEA on macro Kv4.2 currents co-expressed with the DPP10a and Kv4.2 construct with the N-terminal occlusion domain deleted (Kv4.2/2Δ40). Internal TEA reduced the peak current Kv4.2 by ~46% and significantly slowed the time constant of fast inactivation [69]. The competition between the DPP10a N-terminal occlusion domain and TEA was further bolstered by the fact a direct proportionality between the fold-change in the fast inactivation time constant against the fold-change in the peak current as the concentration of TEA is increased (from 5 mM to 10 mM), as indicated by the slope of a linear regression (1) and corresponding intercept (0). Moreover, exposure of Kv4.2/2Δ40 + DPP10a-expressing cells to elevated external K^+^ (98 mM) increased the rate of recovery from inactivation of the channels (Jerng et al., 2009), suggesting external K^+^ relieves block of the DPP10a N-terminal occlusion domain (MNQTA), which is necessary for recovery from inactivation [69]. Together, these experiments provided clear evidence that DPPLs provide the ternary Kv4 complex with fast inactivation via the N-terminal MNQTA domain, with pore-blocking features analogous to N-type inactivation.

### 2.5. DPP6 Accelerates Recovery from Inactivation and Enhances Low-Voltage Availability of Kv4 Channels

The ability of Kv4 channels to recover from inactivation on a millisecond time scale distinguishes them from other channels mediating I_A_. It is well established that the presence of DPP6 in the complex significantly accelerates channel recovery from inactivation [10,50,69]. The modulation of channel transitions between the conducting, open state and an inactivated state underlies their ability to modify this property. The findings that recombinant Kv4 channels exhibit macroscopic inactivation that can be fit with the sum of three exponentials suggested that an overlap in inactivation mechanisms may occur in time, with channels existing in various inactivated states [65,66,72,73]. However, that recovery from inactivation can reliably be fit by a single exponential, with recovery kinetics nearly identical with strong to moderate depolarizing test pulses, favors the notion that these channels preferentially enter a single inactivated state prior to recovery [74]. Overwhelming evidence, including the steep voltage-dependence of steady-state inactivation kinetics, suggests that this inactivated form is adopted from a closed state [55,66,74,75]. As stated previously, KChIPs in the Kv4 complex inhibit rapid inactivation from an open-state via sequestration of the pre-blocking domain in the N-terminus. Confirmation that this likely prevents open-state, N-type inactivation from being the final state adopted prior to recovery was provided by Bahring et al. [66,76], as deletion of the Kv4 N-terminus (2Δ40) did not alter recovery from inactivation. This suggested other factors, which contribute to fast inactivation as well as the stability of channels in their closed state, as important determinants of recovery kinetics. The studies referenced previously provide convincing evidence that DPP6 confers the Kv4 complex with fast inactivation properties that allow channels to quickly reach a pre-open, closed state [32,69]. It is likely that DPP6 interaction with the Kv4 voltage-sensor S1 and S2 transmembrane domains through its transmembrane domain facilitates modulation of open to closed-state dynamics [11,18,51,52,75].

Insights into this hypothesis were provided by Dougherty et al. [77]. Here, they engineered a charybdotoxin (CTX)-sensitive Kv4.2 channel, which blocked K^+^ permeability and enabled the observation of gating currents (I_g_)—manifestations of the gating charge (Q) movements. These currents are capacitive transients that derive from outward and/or inward movements in the voltage sensors (Dougherty et al., 2006). They co-expressed the engineered Kv4.2 channel with or without DPP6s and identified that the I_g_ exhibited significantly faster ON/OFF kinetics in response to step depolarizations in the presence of DPP6s, with rapid Q mobilization. This suggested that DPP6s influenced the relative stability of various conformations of the voltage sensors through changes in the membrane environment around the channel’s core [77]. A follow-up study confirmed that Q immobilization is correlated with the Kv4 closed-state, suggesting that modulation of the Q movements is indicative of DPP6′s ability to promote transitions to the closed, inactivated state from pre-open and open states [75,78]. Additional experimental evidence was provided using a pre-pulse protocol at low voltages where ~50% of channels are inactivated under steady-state condition [32]. DPP10 and DPP6s accelerated pre-open inactivation by a robust 7-fold and 3–4-fold, respectively [32]. Therefore, through its N-terminus and transmembrane domains, it is likely that DPP6 permits the rapid adoption of closed-state inactivation following opening and alter the stability of the channel in this state, rendering them ‘receptive’ to opening upon depolarization.

The interaction between DPP6 and the Kv4 voltage sensors also underlies their ability to modulate the voltage-dependency of channel activation and inactivation. DPP6 appears to foster channel transitions more readily in their presence. Bahring et al. [66], Jerng et al. [32], and Dougherty et al. [77] used kinetic modeling to describe Kv4 channel transitions between open and closed states. From the closed state, channels may enter an open state (conducting) or inactivated state with membrane potentials at subthreshold voltages [75]. Once inactivated, the voltage sensor is immobilized in a stable conformation and resistant to depolarization [77]. This reluctance to open is alleviated by the presence of DPP6, which alters the stability of the closed stated and allows for transient, low-voltage depolarization to permit channel opening. Further, the electromechanical interaction between DPP6 and the S1–S2 domains of the Kv4 voltage sensor is likely to reduce the probability of channel inactivation from the closed state, thus promoting the subthreshold availability of Kv4 channels and relative hyperpolarizing shift in the voltage-dependence of activation [75,77]. Structural observations have also alluded to the potential for DPP6 to alter the movement of the S4-S5 linker in Kv4 during inactivation and recovery from inactivation [75].

### 2.6. DPP6 Regulation of Kv4 Impacts Membrane Excitability and Synaptic Plasticity

The collective effects of DPP6 on I_A_ leads to robust control of important neuronal functions. In all, the investigations described above, undertaken in expression systems, demonstrate how DPP6 might augment Kv4 channel regulation of membrane excitability. However, there was limited evidence of DPP6 regulation of I_A_ in neurons. Kim et al. [79] began answering this question with a study examining DPP6 regulation of AP firing properties in hippocampal pyramidal neurons in organotypic slice cultures. They used viral mediated siRNA knockdown of endogenous DPP6 to examine its role in neuronal sub and suprathreshold membrane properties. Consistent with observations in expression systems, knockdown of DPP6 caused a depolarizing shift and steepening of the I_A_ voltage-dependence of activation and steady-state inactivation, a decrease in the rate of inactivation at low voltages, and a slowing in the rate of recovery from inactivation as measured in outside-out somatic patches. While these changes would suggest DPP6 knockdown may enhance neuronal excitability by suppressing low-voltage Kv4 activation, they found a contradictory decrease in subthreshold excitability in whole cell current-clamp recordings. Specifically, DPP6 knockdown decreased input resistance, which correlated with an extension in latency to AP firing and increased threshold in response to a current injection in a voltage range where Kv4 channels generally mediate a shunting effect. Moreover, loss of DPP6 also impacted membrane excitability at suprathreshold potentials. Namely, siRNA DPP6 neurons displayed APs with increased half-widths and decreased after-hyperpolarization amplitudes and a slight reduction in AP firing frequency relative to control. A combination of current-clamp recordings and computer simulations revealed the likelihood that the surprising decrease in subthreshold excitability with DPP6 knockdown results from a complex interplay with additional voltage-gated channels, including HCN and Na^+^ channels. The extension of the I_A_ window current via a depolarizing shift in steady-state inactivation permits an increase in the availability of channels near rest. Together with a steepening of the voltage-dependence of activation curve, this enables more channels to conduct hyperpolarized to threshold, delaying Na^+^ channel activation and increasing AP latency. Although these findings were relatively unexpected, the authors, nonetheless, identified a crucial role for DPP6 in regulating neuronal excitability through its modulation of Kv4 channel activity. This also emphasized the need to pursue additional studies of DPP6-Kv4 regulation of neuronal physiology given the complexity of channel activity in a native environment.

While somatic Kv4 channels can impact neuronal firing modes relevant to neural computations, their privileged distribution in dendrites suggests that their control by DPP6 may be more evident and impactful in these subcellular regions [38,45,80,81]. Hoffman et al. (1997) [38] established that I_A_ is non-uniformly expressed in the primary apical dendrites of rat hippocampal CA1 pyramidal neurons, with density increasing with distance from the soma. This gradient significantly enhances the capacity for apical dendrites to filter distal synaptic inputs and suppress backpropagating action potentials (bAPs), as they serve as dendritic ‘shock absorbers’ [38]. As a result, the ability of Kv4 channels to impact membrane potentials in dendrites and dendritic spines drastically impacts the recruitment of the molecular drivers of synaptic plasticity, including NMDA channels and their downstream effectors [82,83]. Therefore, identifying the underlying mechanisms establishing this subcellular distribution was imperative.

Sun et al. [20] identified DPP6 as a critical factor for setting the apical dendritic gradient. They used cell-attached dendritic patch-clamp recordings in conditional DPP6-KO and WT mice to determine the role of this auxiliary subunit. They identified that DPP6-KO mice lacked the apical dendritic I_A_ gradient that was prominent in WT [20]. At approximately 100 µM from the soma, peak amplitude of the transient I_A_ was reduced relative to WT, which deviated further with distance (up to 240 µM). Somatic and proximal dendritic I_A_ (<80 µM) remained unchanged, suggesting the subcellular compartmentalization of DPP6 protein modulation of the Kv4 function. Additionally, the voltage-dependence of activation was shifted to a more depolarized potential and recovery from inactivation was delayed in remaining currents measured in the distal dendrites, reminiscent of Kv4 currents measured in the absence of DPP6 in heterologous expression systems. Functionally, the loss of DPP6 significantly increased dendritic calcium electrogenesis and boosted overall dendritic excitability by increasing the amplitude of bAPs [20].

Additionally, the Kv4-mediated regulation of dendritic excitability impacted spike-time dependent plasticity (STDP). DPP6-KO mice displayed enhanced LTP in response to a theta-burst pairing protocol relative to WT mice. Because the coincident detection of bAPs and synaptic potentials is crucial in dictating not only the magnitude of plasticity, but also the direction, the identification of a significant impact on STDP-LTP in DPP6-KO mice might have been anticipated. The examination of membrane potentials within the spike-timing window revealed the integral of membrane depolarization between APs following excitatory post-synaptic potentials was enhanced in DPP6-KO mice, most likely indicative of an augmented Ca^2+^ spikes [20]. The increased Ca^2+^ influx during the induction of STDP is likely responsible for the extension of the window to induce potentiation as well as its subsequent maintenance. Recently, such Ca^2+^ spikes have been shown to rapidly induce plasticity in CA1 hippocampal neurons to form de novo “place cells”, which fire at a high rate whenever the animal is in a specific location in the environment [84]. Even subtle changes in dendritic and dendritic spine Ca^2+^ dynamics are capable of driving differential second messenger cascades integral in synaptic potentiation and/or depression [85].

Furthermore, DPP6′s impact on I_A_ along the length of the apical dendrites is likely to have developmental effects on hippocampal circuit formation and maintenance. I_A_ magnitude regulates the composition of NMDA receptors through the modulation of the developmental NMDA GluN2B/A switch [82]. This switch is integral in circuit maturation and, as a result, has broad impacts on learning and memory [86]. Therefore, this study exhibited that DPP6 is critical in mediating dendritic Kv4 function, which broadly contributes to the information processing capacity of hippocampal neurons.

## 3. Novel Roles of DPP6

### 3.1. Promoting Growth and Stabilizing Dendritic Filopodia

As described above, DPP6 had a well-established role as an auxiliary subunit influencing the expression and function of Kv4 potassium channels. More recently, novel functions of DPP6 have been brought to light. The results from Foeger et al. [21] first suggested potential functions separate from Kv4.2. They found that, unlike KChIPs, both DPP6 and DPP10 traffic to the membrane independently of Kv4. In cell lysates null for Kv4.2 and Kv4.3, it was found that DPP6 expression remained unchanged compared to WT, whereas practically all KChIP expression was eliminated. Foeger et al. found that the localization of Kv4.2 to the cell membrane was not significantly increased when the cells also expressed DPP10-YFP compared to Kv4.2 alone. DPP6-YFP with Kv4.2, however, showed an increase in both Kv4.2 localization to the membrane and Kv4-mediated current densities.

**Figure 3:**
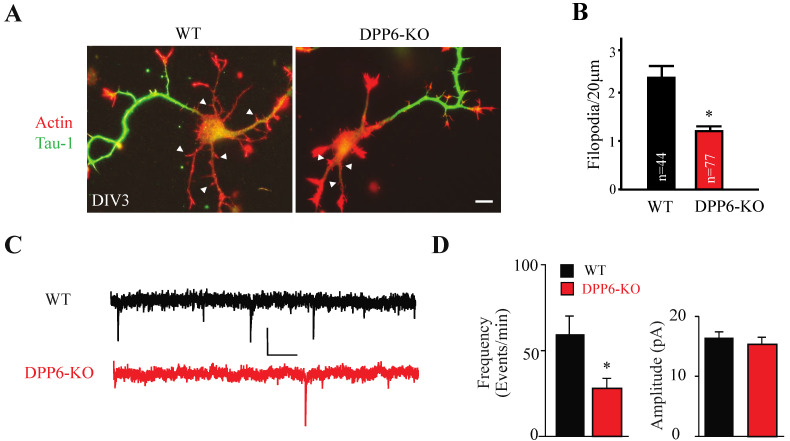
**Dendritic filopodia formation is reduced in DPP6-KO hippocampal neurons and rescued by DPP6 expression.** (**A**,**B**) DIV3 WT or DPP6-KO hippocampal neurons visualized for actin filaments with TRITC-phalloidin (red) and for axons with marker Tau-1 (green). The density of immature dendritic filopodia (arrowheads) is decreased in the DPP6-KO neurons compared to the WT. Scale bars = 10 μm. (**C**) Sample mEPSC traces from WT and DPP6-KO cultured hippocampal neurons recorded at DIV14 and 15. Scale bars: 10 pA, 250. (**D**) Average mini frequency is decreased in DPP6-KO versus WT cultured hippocampal neurons, whereas amplitude is not affected. Error bars represent the mean ± SEM. * *p* < 0.05. From Figure 1 and Figure 3 in Lin et al., 2013 [22].

The following year, Lin et al. (2013) [22] found, independent of its role as a Kv4 auxiliary subunit, that DPP6 was identified to promote and maintain the growth and stability of filopodia, finger-like projects extending from both dendrites and axons, which help create synaptic connections as well as serve as precursors to dendritic spines [87]. In a series of experiments, Lin et al. showed that DPP6 is required for filopodia to develop normally and properly transition into dendritic spines (Figure 3) [22]. DPP6′s function in filopodia regulation ultimately leads to a critical role in behavior and brain development [23]. This may, at least in part, explain DPP6′s association with neurodegenerative diseases and cognitive disorders such as amyotrophic lateral sclerosis (ALS), dementia, Tourette’s syndrome, microcephaly, and autism spectrum disorder (ASD) in addition to other illnesses or disabilities [24,25,26,27,28].

To show the connection between filopodia and DPP6, Lin et al. [22] first quantified the density of immature dendritic filopodia in cultured hippocampal neurons from DPP6-KO and WT mice at DIV3. There was a ~45% reduction in DPP6-KO compared to WT, and a significant decrease in immature dendritic filopodia density (Figure 3A,B). When expressing GFP-tagged DPP6 (DPP6-GFP), there was a significant increase in immature dendritic filopodia compared to the expression of GFP alone. To better understand how DPP6 influenced the number of filopodia, they measured filopodia in live imaging experiments. The average life span of existing filopodia was significantly reduced in DPP6-KO neurons. After transfecting DPP6-GFP into DPP6-KO neurons at P0, filopodia stability was rescued. While monitoring the stability of filopodia, the number of new filopodia formed was also counted, showing that DPP6-KO neurons produce ~60% fewer filopodia than WT neurons. Transfecting the DPP6-KO neurons with DPP6-GFP increased the formation of new filopodia three-fold compared to the GFP control, effectively rescuing the deficiency.

Given that dendritic filopodia transform into dendritic spines, Lin et al. [22] also identified any changes in spine density and function. Using immunofluorescence, they were able to visualize post-synaptic dendritic spines, showing DIV14 cultured DPP6-KO hippocampal neurons had a 30% reduction in dendritic spine density compared to WT. Again, when transfecting DPP6-GFP at P0, the density of dendritic spines was rescued at DIV14 compared to the GFP control. Recording miniature excitatory post-synaptic currents (mEPSCs) from cultured WT and DPP6-KO neurons at DIV14 showed no change in the amplitude of mEPSCs, but a decrease in the frequency, confirming DPP6 modulates spine formation and subsequently synapse number in hippocampal neurons (Figure 3C,D).

To better understand DPP6′s role in synaptogenesis, researchers used RNA interference in E18 rat hippocampi to inhibit the expression of DPP6 (siDPP6). When compared to a control RNAi (siCtrl), there was no difference in the number of filopodia between siDPP6 DPP6-KO and siCtrl DPP6-KO. Between DIV7, 14, and 21, it was observed through immunostaining that cultured WT hippocampal neurons displayed a decrease in density of filopodia, while dendritic spines and synapses increased at the same rate. siDPP6 showed a significantly reduced number of filopodia at all three days as well as decreased dendritic density compared to siCtrl. The mEPSC amplitude was unchanged, whereas, as in previous experiments, mEPSC frequency was decreased [22]. The difference in frequency supports the notion that fewer synapses were formed due to decreased filopodia acting as precursors.

Biocytin staining showed DPP6 loss altered dendritic morphology in hippocampal CA1 pyramidal neurons from both adult WT and DPP6-KO mice. Sholl analysis revealed a simpler dendritic branching pattern in neurons from DPP6-KO mice compared to WT controls. The decrease in functional excitatory synapses in DPP6-KO CA1 neurons may then be due to their sparser dendritic arborization in addition to their reduced spine density per unit length of remaining apical dendrites. DPP6′s effect on the developing brain is likely during a critical period given the reduced adult brain weight, simplified dendritic arbors, and decreased synaptic connections observed in adult DPP6-KO mice, which can affect cognitive processes [22]. 

### 3.2. Cell Adhesion

Crystalline structure shows that DPP6, like other DPPs, has a large extracellular domain. Lin et al. (2013) found that this extensive extracellular domain interacts with proteins in the extracellular matrix (ECM). Through these contacts, DPP6 acts similarly to family member DPP4/CD26 to promote cellular adhesion. Specifically, Lin et al. found that DPP6 interacts with the arginine-glycine-aspartate (RGD) motif [22]. RGD motifs are important integrin-binding regions of ECM proteins like fibronectin, regulating the efficiency of cellular adhesion [88]. This finding further supports DPP6′s role in filopodia formation and stability through integrin binding. Filopodia rely on cellular adhesion to local ECM proteins for assistance when extending [89]. DPP6′s interaction with ECM proteins therefore provides stabilization and connections to promote filopodia growth and maintenance. 

### 3.3. Recognition, Learning, and Memory

Lin et al. (2018) continued the study of DPP6 synaptic function to determine how altered synaptic development affected learning and memory. Using various behavioral measures such as the Morris water maze, T-maze, fear conditioning, spatial and novel object recognition, and open field and home-cage locomotor activity, they found that adult DPP6-KO mice had difficulty with spatial memory compared to that of WT mice [23]. When tasked with finding a submerged platform in the water maze, the DPP6-KO mice took longer, despite having the same average swimming speed as WT. In the T-maze, with rewards on altering sides each trial, DPP6-KO mice did not show improving success rate during training as WT littermates do. DPP6-KO mice also had memory deficits observed in fear conditioning experiments. Using classical conditioning, it was found that the DPP6-KO mice had significantly decreased freezing responses compared to WT. In the novel object recognition task, DPP6-KO mice showed no preference for the novel object compared to WT mice [23]. In all tests, the DPP6-KOs had delayed times for recognizing familiar tasks and objects, showing that this auxiliary subunit plays a role in these learning and memory through its role in synapse formation and/or as an auxiliary subunit to Kv4 channels.

## 4. Clinical Manifestations of DPP6 Malfunction

The multifaceted roles of DPP6 in regulating synaptic maturation, cell adhesion, neuronal excitability, synaptic plasticity and learning and memory underscores its importance in normal brain function. Impairments in the connectivity and balance in excitability of neurons and circuits in various brain regions underlies both psychiatric and motor deficits in disease. It may not be surprising, therefore, that DPP6 appears as a contributor to the pathogenesis of a host of neurological disorders (Table 1). Recent analyses have indeed implicated DPP6 in the progression of multiple diseases affecting the nervous system. Here, we briefly discuss these findings.

### 4.1. Intellectual Disability

Recently, missense variations of DPP6 have been identified in human patients with microencephaly. When researching patients with autosomal dominant microcephaly and intellectual disability (ID), it was found that DPP6 expression was reduced due to presence of a missense variation or deletion at the 7q36.2 loci in multiple subjects. The result of these alterations leads to reduced rodent brain weight as well as learning disabilities and the autosomal dominant microcephaly [25].

Liao et al. [25] confirmed that a copy number variation of the DPP6 gene in patients is associated with autosomal dominant microcephaly and variable ID. The analysis was performed on DNA samples from 22 patients with microcephaly using high-resolution, array-based genomic hybridization. Two patients with small de novo deletions in the DPP6 gene were identified. Sequence analysis was also performed in another 50 microcephalic patients. A missense mutation in the DPP6 gene was identified in a family segregating microcephaly and autosomal dominant ID. In mice, knockdown of DPP6 using short hairpin RNA resulted in smaller brains and learning and memory disabilities as assessed by their performance in the Morris water maze compared to WT littermates. However, the particular importance of DPP6 in human developmental brain diseases may be inferred from an earlier study by Dorus et al. showing that DPP6 is one of the nervous system genes that show a faster protein evolution in primates compared to rodents, thus implicating DPP6 in regulating human brain size and behaviors [90].

### 4.2. Dementias

DPP6 was recently shown to be a candidate gene in dementia [28]. Here, Cacace et al. employed a long-read whole genome sequencing technique to identify and validate a chromosomal inversion of 4 Mb segregating at 7q36 in intron 1 of DPP6 from the disease haplotype in a family with autosomal dominant dementia. This locus (7q36) was first identified as a locus for Alzheimer’s disease in 2005 by Rademakers et al. [91], although the involvement of DPP6 in Alzheimer’s disease was not clear in this earlier study. Cacace et al. [28] found significantly higher rare variants of DPP6 in early onset Alzheimer’s disease and frontotemporal dementia (FTD) patient cohorts. They also found evidence for reductions in DPP6 RNA and protein and Kv4.2 protein in the brains from patients carrying rare DPP6 missense variants. Overall, they found that the loss of DPP6 expression and function can have a significant impact in dementia.

Another finding by Zelaya et al. [92] demonstrated an olfactory progressive proteome modulation in Alzheimer’s disease, which shows olfactory dysfunction in up to 90% of patients. They found a specific late reduction in DPP6 in the olfactory bulb of patients with Alzheimer’s disease [92]. This finding provided basic information for understanding the implication of the olfactory bulb in the pathophysiology of Alzheimer’s disease, identifying protein mediators that may be used as potential therapeutic agents or even as candidate biomarkers for its diagnosis and evolution.

**Figure 4:**
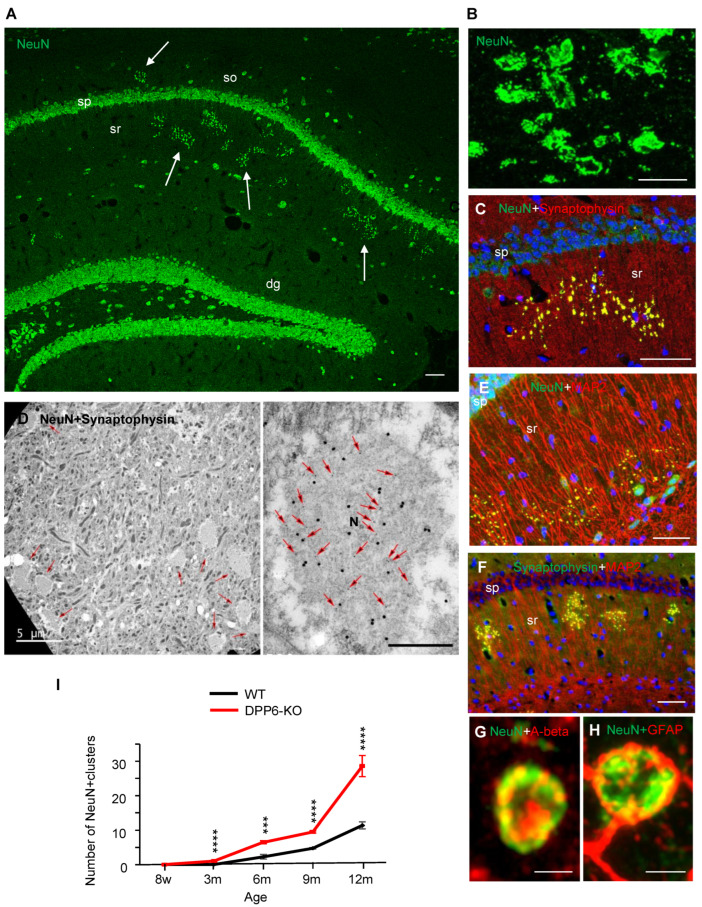
**Novel large clusters of NeuN+ puncta are found in the hippocampus of DPP6-KO mice.** In the CA1 region of the hippocampus of 12-month-old DPP6-KO brain sections, (**A**) immunofluorescence (IF) labeling for NeuN (green) is concentrated not only in the neuronal somatic layer but also in novel structures made of enlarged, variable puncta formed in clusters (arrows). Scale bar = 100 μm, (**B**) higher magnification of single clusters from another section. Scale bar = 5 μm. (**C**) IF for NeuN (green) and synaptophysin (red) show complete colocalization in clusters of the novel large puncta. Scale bar = 50 μm. (**D**) Immunogold labeling for NeuN and synaptophysin in the deep region of the CA1 of the DPP6-KO mouse; 20 nm gold for NeuN is concentrated within the large swellings (arrows in low magnification image on the left; N in high magnification image on the right), where it colocalizes very well with 10 nm gold for synaptophysin (arrows in high magnification image; all 10 nm gold particles in the image are indicated with the arrows. Scale bar = 500 nm). (**E**) IF for NeuN (green) and MAP2 (red) are partly colocalized in the large novel puncta clusters, with MAP2 on the periphery of most NeuN+ puncta and throughout some of them; MAP2 also is evident in the apical dendrites of the CA1 pyramidal neurons that overlap with the clusters. Scale bar = 50 μm. (**F**) IF shows MAP2 (red) and synaptophysin (green) partly colocalized in clusters of puncta. Scale bar = 50 μm. (**G**) IF shows Aβ (red) partially colocalized with the novel NeuN+ (green) structures. Scale bar = 2 µm. (**H**) IF shows that the GFAP+ astrocytes can directly surround the novel NeuN+ large puncta, sometimes as distinct ring-shaped processes. Scale bar = 2 µm. (**I**) Graph comparing the number of novel clusters in the CA1 region of the hippocampus during development from 8-week to 12-month-old mice in WT and DPP6-KO mice. From 8-week to 12-month-old, labeling in aged DPP6-KO mice is significantly increased compared to WT (*** = *p* < 0.001, **** = *p* < 0.0001). Nuclei were counterstained with DAPI in blue. dg, dentate gyrus; so, stratum oriens; sp, stratum pyramidale; sr, stratum radiatum. Lin et al., 2020 [93].

In a recent paper [93], we reported a novel structure in the hippocampal CA1 region that was significantly more prevalent in aging DPP6-KO mice compared to WT of the same age. These structures were also observed earlier in development in DPP6-KO mice. These novel structures appeared as clusters of large puncta that colocalized NeuN, synaptophysin, and chromogranin A and also partially labeled for MAP2, amyloid β, APP, a-synuclein, and phosphorylated tau, with synapsin-1 and VGluT1 labeling on their periphery. Electron microscopy revealed that these structures are abnormal, enlarged presynaptic swellings filled with mainly fibrous material with occasional peripheral, presynaptic active zones forming synapses. Immunofluorescence imaging showed that a number of markers for aging and Alzheimer’s disease were found at higher levels in the novel structures of aging DPP6-KO mice compared to WT. All these results indicate that aging DPP6-KO mice show characteristic symptoms similar to those found in Alzheimer’s disease, i.e., progressive cognitive and learning and memory impairment resulting from synapse loss and neuronal death (Figure 4).

DPP10 may function similarly to DPP6. DPP10 has been associated with bipolar disorder by Djurovic et al. [94], who carried out a genome-wide association study (GWAS) [94]. Chen et al. [95] have observed truncated forms of DPP10 in brain tissue from Alzheimer patients; these truncated forms may lead to the mis-trafficking and aggregation of DPP10 protein observed in tangles and plaques associated with the disease. The presence of DPP10 in tangles and plaques has also been detected in other neurodegenerative diseases, such as diffuse Lewy body disease and frontotemporal dementia [95,96].

### 4.3. Motor Disorders

Amyotrophic lateral sclerosis (ALS) is a neurodegenerative disease characterized by progressive muscular paralysis reflecting degeneration of motor neurons in the primary motor cortex, corticospinal tracts, brain stem and spinal cord. The average survival time is three years from disease onset. No effective treatment is available [97]. The cause of ALS is largely unknown, but genetic factors are thought to play a significant role in determining susceptibility to motor neuron degeneration. Van Es et al. [98] have identified a genome-wide SNP in the DPP6 gene that is significantly associated with susceptibility to sporadic ALS in different populations of European ancestry. They identified single nucleotide polymorphisms (SNPs); rs10260404 is located within intron 3 of DPP6 [98]. Later, this group reported that the same SNP of DPP6 also associated with progressive spinal muscular atrophy, which is related to ALS but is only characterized by loss of lower motor neurons, causing progressive muscle weakness [98,99,100]. However, functional variants of DPP6 have not been discovered, and the risk of mutations in DPP6 are not significantly associated with ALS, according to other reports in different locations of populations [24,101,102,103,104]. These results showed that SNPs of DPP6 only associate with a limited number of ALS cases; other studies have reported that rare copy number variations (CNV) of DPP6 have been associated with ALS [105,106].

DPP6 is a candidate gene for neuroleptic-induced tardive dyskinesia [107]. Tardive dyskinesia (TD) is the involuntary movement of the tongue, lips, face, trunk and extremities that occurs in patients who are undergoing long-term treatment with antipsychotic medication. By using a genome-wide SNP array in schizophrenia patients with or without treatment-resistant TD, they showed that DPP6 is a promising association gene with the SNP located in intron-1 and associated with decreased DPP6 expression in the human postmortem prefrontal cortex. Interestingly, in a mouse model, DPP6 expression is increased in the prefrontal, striatal, hippocampal and ventricular midbrain brain regions after 50 weeks’ long-term administration of haloperidol, which is an antipsychotic drug that reduces the level of excitement in schizophrenia, Tourette’s syndrome and severe behavioral problems in children. These findings indicated that Kv4/DPP6 is involved in the neuroleptic-induced TD through their activity regulation in dopamine neurotransmission and the resulting hypersensitivity to dopamine.

### 4.4. Neurodevelopmental and Psychiatric Disorders

Autism spectrum disorder (ASD) is a range of neurodevelopmental disorders starting in early childhood and is characterized by impairments in communication and reciprocal social interaction and presence of restricted and repetitive patterns of behavior. ASD is a complex non-Mendelian disorder. It is caused by a combination of rare de novo and inherited copy number variants (CNV) as well as common variants in at least several hundred genes [108,109,110,111,112,113,114]. Copy number variants including the deletion, duplication, translocation, and inversion of chromosomes have been identified and play a significant role in the etiology of ASD. Marshall et al. [108] performed a genome-wide assessment for structural abnormalities in 427 unrelated ASD cases utilizing single-nucleotide polymorphism microarrays and karyotyping. They discovered 277 unbalanced CNVs in 44% of ASD families, and these were absent from the 1652 controls. De novo CNVs were found in ~7% and ~2% of idiopathic families having one child, or two or more ASD siblings, respectively. They identified numerous new ASD loci at DPP6 and DPP10 genes, which have been first reported to have either CNV gains or losses in unrelated individuals at the same locus. Put together with other findings of SHANK3, NLGN4, and NRXN1-PSD genes, all the genes have functions associated with the postsynaptic density, as well as with intellectual disability and dysregulation of gene expression.

More studies have reported that DPP6 or DPP10 are risk genes in ASD, using various different assays for identification [113,115]. Furthermore, Bock et al. [116] reported that a DPP6 mutation at Arg322Cys was present in two sibling children with autism and showed paternal inheritance in their father and grandfather; this Arg322Cys mutation, which is in the eight-bladed β-propeller domain of DPP6, may impair the function of DPP6 [116]. Maussion et al. [117] reported a case of synergism. An individual who had a sensory processing disorder, apraxia, and autism, and was from a family with a variable psychiatric disorder, had mutation and disruptions in both DPP6 and LRRC4C genes, even though the variant by itself was not cause the autism. It suggests both K+ channel regulation and axon guidance are important in development [117]. These various findings may help future diagnosis and treatment of ASD.

The mutation of DPP6 has been reported in Gilles de la Tourette syndrome (GTS) (Prontera et al., 2014). GTS is a neurodevelopmental disorder, highly heritable and childhood-onset, that is characterized by several motor and phonic tics. Tics usually develop before 10 years of age, exhibit a waxing and waning course, and typically improve with increasing age [118]. Prontera et al. have identified a heterozygous microdeletion at the first exon of DPP6 in a boy with GTS as well as the boy’s father and paternal uncle, both of whom were diagnosed with tic disorder and ADHD (attention deficit hyperactivity disorder). mRNA levels of DPP6 were also decreased in the boy’s blood cells [26,119].

Recently, Naujock et al., 2020 screened induced pluripotent stem cell (iPSC) lines from skin fibroblasts from healthy donors and patients diagnosed with idiopathic schizophrenia (SZ) [120]. Human iPSCs were induced into cortical neurons both as monolayers and as three-dimensional spheroids. RNA sequencing revealed that mRNA transcripts encoding DPP6 were increased in cortical neurons from SZ iPSCs both models. As would be expected, given DPP6′s role in augmenting Kv4 conductance, decreased neuronal activity was found in cultures from SZ neurons. These results were reversed in experiments aimed at downregulating either DPP6 or Kv4.2. DPP6 also has been reported as a putative candidate gene for schizophrenia with very rare CNVs [121].

**Table 1:** DPP6-related diseases.

Disease	Major Symptoms	Genome	Refs
Dementia	Forgetfulness, limited social skills, and thinking abilities	Higher rare variants	[28,93]
Alzheimer’s disease	Memory loss and confusion		[91,92,93]
Amyotrophic lateral sclerosis (ALS)	Progressive muscular paralysis	SNP-rs10260404 is located within intron 3, CNVs	[98,100,105,106]
Spinal muscular atrophy (PMA)	Progressive muscle weakness	SNP-rs10260404 is located within intron 3	[99]
Neuroleptic-induced tardive dyskinesia (TD)	Involuntary movement	SNP in intron-1 of DPP6 in East Asian	[107]
Autism spectrum disorder (ASD)	Difficulty with communication, social interactions, obsessive interests, and repetitive behaviors	CNV gains or losses both DPP6 and LRRC4C	[108,115,116,117]
Microcephaly and intellectual difficulty		Rare CNVs	[25]
Gilles de la Tourette syndrome	Several motor and phonic tics	Heterozygous microdeletion at the first exon of DPP6	[26]
Schizophrenia		Rare CNVs	[120,121]

## 5. Future Directions and Open Questions

It has become clear that DPP6 maintains a crucial role in normal brain function through its association with Kv4 channel complexes and, independently, in synaptic maturation. Parsing its function in both of these contexts will be required to fully understand its role in disease. While much work remains, the mechanisms driving dynamic regulation of the Kv4.2 complex are beginning to be unraveled. Hu et al. [122] investigated the regulatory control of the Kv4-DPP6 complex through a p38 kinase-Peptidyl-prolyl cis-trans isomerase NIMA-interacting 1 (Pin1) cascade. By analyzing phosphorylation sites and studying structural rearrangements using mutant Pin1 it was found that Kv4.2-Pin1 binding is direct on the C-terminus of Kv4.2 at site T602, and, more prominently, at T607. After confirming the association of p38 kinase and Kv4.2, mutant p38 revealed that p38 is the key kinase in the phosphorylation of Kv4.2 at the T607 site in mice hippocampi, allowing Pin1-Kv4.2 interaction.

Given DPP6′s role as an auxiliary subunit, Hu et al. investigated p38 and Pin1′s regulation of Kv4.2-DPP6 association. Seizure induction, followed by co-IP, showed that p38 phosphorylation followed by Pin1 binding lead to DPP6 dissociation from Kv4.2. Additionally, Pin1 was highlighted as a regulatory control of neuronal excitability through its enzymatic action on Kv4.2. CA1 pyramidal neurons from mice expressing a Kv4.2 T607 mutation (Kv4.2TA) to prevent Pin1 binding were not affected by pharmacologically blocking p38 or Pin1, whereas WT neurons showed increased I_A_ currents in response to this pharmacological blockade [122]. The presumed mechanism of this increase in I_A_ is the prevention of p38-Pin1-mediated dissociation of Kv4.2 and DPP6. Similar blockade of Kv4.2-DPP6 dissociation was also shown to reduce kainic acid induced seizure intensity in mice [123].

To determine whether this increase in I_A_ current affects cognitive processes, Hu et al. compared Kv4.2TA and WT mice in learning behavioral tests. In all cases, Kv4.2TA preformed as well as WT mice in initial learning. However, in reversal learning tests, the mutant Kv4.2TA mice outperformed WT mice. These results show that when the fraction of Kv4.2 channels in complex with DPP6 is increased, their cognitive flexibility is enhanced [122]. To confirm the cellular function that contributed to the boosted cognitive flexibility seen in the reversal learning, future studies involving more detailed long-term synaptic plasticity measures in concert with imaging may be employed to ascribe a cellular and circuit correlate to the phenotype. It is plausible that activity-dependent/dynamic regulation of DPP6-Kv4.2 association and turnover may impart synapse state-dependent changes, altering the expression and consolidation of synaptic plasticity in a bi-directional manner.

The work from Hu et al. [122,123] identifies a novel post-translational cascade impacting Kv4.2 channel complexes. This adds to a growing list of modifications affecting Kv4 complex membrane localization and conductance. The novelty of the cognitive phenotype observed in this context suggests a unique mechanism not seen in other Kv4 mouse models. Loss of function mutations and overexpression of the Kv4 alpha-subunits have been shown to affect initial learning, but the state-dependence seen here is unique. While this may signal that dynamic regulation of the complex over developmental time periods may impart distinct synaptic changes relative to constitutive loss/gain of function, additional mechanisms may be at play. Might the p38-Pin1 cascade preferentially target certain pools of Kv4 complexes? A fundamental question that has yet to be fully addressed is whether endogenous Kv4 complexes exist in the absence of DPP6. If so, what proportion of complexes are absent or containing DPP6? Further, do select KChIP isoforms associate with certain Kv4/DPP6 pools of complexes? As described above, complexes absent DPP6 or in association with various KChIPs would display quite dramatic differences in voltage-dependence, gating kinetics and conductance, impacting local membrane potential and synaptic/dendritic integration in distinct ways. Additionally, complexes with disparate associations may be subject to differential post-translational modification and/or Ca^2+^ regulation [64]. It is possible various activity patterns within neural circuits lead to the turnover of select Kv4 complexes via the activation of a litany of second messenger signaling cascades.

It is notable that somatic patch-clamp recordings of I_A_ display different kinetics from more distal apical dendritic recordings in outside-out patches in CA1 hippocampal pyramidal neurons [20,38,122]. While the increased density of the channels along apical dendrite and presence of competing currents from additional voltage-gated channels may partially explain these differences, a subcellular gradient in auxiliary subunits supporting the complexes and/or post-translational modifiers, as described above, may contribute. Immunohistochemical procedures have provided insights [45,80,81]; however, more precise measures are needed. Highly selective pharmacological tools could prove useful in assessing the stoichiometry of Kv4 complexes in association with auxiliary subunits, including DPP6. One such tool has been characterized and put into practice. AmmTX3, a member of the scorpion toxin alpha-KTX15 family, has been identified as a selective inhibitor of Kv4. When studying the blockage of Kv4 using AmmTX3, Maffie et al. showed that it is required for DPP6 or 10 to be in complex with the channel [124]. Given the efficacy and steepness of the concentration dependency of I_A_ block by AmmTX3, this tool may be used to assess the proportion of Kv4 channels in association with DPP6 by means of sensitivity to the drug [122]. It would be useful to further research to discover other blockers/labels that select for distinct pools of Kv4 complexes.

In addition to subcellular distribution of DPP6 and Kv4 complexes, investigation into cell specific expression of their isoforms may point to the differential regulation of excitability in various brain regions. The majority of studies to date focus on DPP6-Kv4.2 in hippocampal CA1 pyramidal neurons, but to fully appreciate DPP6 contributions to neural circuit function in normal and pathogenic states, the expression pattern of isoforms is required. Furthermore, the cell-specific expression of various DPP6 isoforms in concert with Kv4 alpha-subunits within regions of the brain, including the hippocampus, are required to assess the full potential of DPP6 modulation of I_A_ within circuits. Increased single cell sequencing in neuronal subtypes has provided pertinent information and more studies will assuredly follow. When looking at KChIPs 1–4 and DPPLs, Olah et al. [125] uncovered that these proteins and subunit splice variants influenced various outcomes of Kv4 channels in CCK+ interneurons in CA3 region of the hippocampus. The DPP10 isoform that was found in Olah et al.’s analysis of transient outward rectifying CCK+ cells (TOR) and regular spiking (RS)cells was predominantly the DPP10c variant, whereas only DPP6L was found in RS cells. Both DPP6S and DPP6L were found in TOR cells; however, DPP6S was more abundant [125]. Disparate combinations of these alternate splice variants lead to different firing properties of the cell types, suggestive of significant divergence in the control of Kv4-mediated I_A._ [125]. Alternatively, Chittajallu et al. (2020) reported an additional subset of hippocampal interneurons in CA1, neurogliaform cells (NGFC), which display prominent transient Kv4-mediated I_A._ In situ hybridization with RNA scope platform revealed the likelihood of Kv4.2 or Kv4.3 as the predominate carriers of I_A_ in these cells [126]. Interestingly, the abundant expression of both DPP6 and DPP10 was present in these cells [126]. DPPL-Kv4.2 modulation of neuronal physiology was also present in this interneuron population as AmmTX3 exposure removed a delay to spiking, leading to increased excitability. This enhancement in excitability was also seen in a novel intrinsic plasticity termed short term potentiation of somatic depolarization driven excitability (STP-SE). STP-SE is more easily produced in an environment when I_A_ is inhibited. These results indicate that there is activity-dependent modulation of the DPPL-Kv4 complex occurring at some level in an interneuron population that exerts strong inhibition of distal apical dendrites of hippocampal pyramidal neurons [126]. Future studies may center on activity-dependent post-translational modification of I_A_ in the context of STP-SE, which could reveal novel mechanisms involving other DPPL-Kv4 complexes.

Investigation into DPP6 distribution independent of Kv4 is also in critical. It seems likely that there is a separate pool of DPP6 proteins that perform functions in synapse formation. What, if any, are the binding partners for this pool of DPP6 complexes? Further, are specific isoforms more readily absent from Kv4 complexes, and are these most likely to be involved in the Kv4-independent roles? Structural observations have revealed a large extracellular domain of DPP6 that could feasibly serve as an interacting partner with a transsynaptic partner(s) of unknown identity [75]. This possibility would be of especial intrigue in the context of neurodevelopmental disorders. As detailed above, DPP6 is a multifunctional protein with considerable impact on brain development and function. That said, researchers and clinicians have much yet to discover and elucidate concerning DPP6’s regulation and function in the coming years.

## Data Availability

Not applicable.
